# Polycomb Repressor Complex 2 in Genomic Instability and Cancer

**DOI:** 10.3390/ijms18081657

**Published:** 2017-07-30

**Authors:** Zoe Veneti, Kalliopi K. Gkouskou, Aristides G. Eliopoulos

**Affiliations:** 1 Molecular and Cellular Biology Laboratory, Division of Basic Sciences, University of Crete Medical School, 71003 Heraklion, Greece; veneti@imbb.forth.gr; 2 Embiodiagnostics Ltd., 71202 Heraklion, Greece; gkouskoukal@gmail.com; 3 Department of Medical Laboratories, Faculty of Health and Caring Professions, Technological and Educational Institute of Athens, 12243 Athens, Greece; 4 Institute of Molecular Biology and Biotechnology, Foundation for Research and Technology Hellas, 70013 Heraklion, Greece

**Keywords:** polycomb, polycomb repressor complex 2, enhancer of zeste homolog 2, DNA damage response, diet, inhibitors

## Abstract

Polycomb repressor complexes PRC1 and PRC2 regulate chromatin compaction and gene expression, and are widely recognized for their fundamental contributions to developmental processes. Herein, we summarize the existing evidence and molecular mechanisms linking PRC-mediated epigenetic aberrations to genomic instability and malignancy, with a particular focus on the role of deregulated PRC2 in tumor suppressor gene expression, the DNA damage response, and the fidelity of DNA replication. We also discuss some of the recent advances in the development of pharmacological and dietary interventions affecting PRC2, which point to promising applications for the prevention and management of human malignancies.

## 1. Introduction

Polycomb group (PcG) proteins were first identified as repressors of homeotic (*Hox*) genes, which ensure the fidelity of developmental patterning in *Drosophila melanogaster* [1]. Since then, they have been found in organisms as diverse as insects, mammals and plants, where they are involved in the repression of gene expression through chromatin remodeling. These proteins are still a subject of intense investigation, predominantly due to their fundamental role in developmental processes such as cell fate and lineage decisions, cellular memory, stem cell function, and tissue homeostasis [2].

Most PcG proteins are part of transcriptional-repressive complexes, termed PRCs. Two major polycomb repressor complexes (PRCs) have been identified, PRC1 and PRC2. Members of PRC2 are highly conserved between plants and animals, including unicellular eukaryotes such as alga Chlamydomonas and yeast *Cryptococcus neoformans*, whereas PRC1 proteins are less well conserved. In mammals, PRC2 comprises three core PcG components: enhancer of zeste 2 (EZH2) or its close homolog EZH1, suppressor of zeste 12 (SUZ12), and embryonic ectoderm development (EED). As part of the PRC2 complex, EZH2 and EZH1 display catalytic activity, inducing mono, di- and trimethylation (me3) of histone 3 at lysine 27 (H3-K27). The H3-K27me3 mark can in turn act as a docking site for the chromobox-domain (CBX) protein subunits of PRC1 complexes which, once assembled, catalyze the monoubiquitination of H2A on K119 (H2A-K119Ub). The aforementioned sequential recruitment of PRC2 and PRC1 creates polycomb chromatin domains that facilitate polynucleosome compaction. This, in turn, leads to transcriptional repression by reducing the accessibility both of transcription factors and chromatin-remodelling machineries, such as SWI/SNF. However, whether PRC-associated chromatin marks H3-K27me3 and H2A-K119Ub are required for chromatin compaction, or lead to other functional outputs, remains unclear. In *Drosophila*, polycomb proteins are additionally involved in longer-range chromatin contacts, which suggests effects of PcG proteins on higher-order chromatin organization (reviewed in Di Croce and Helin [3]).

Various developmental pathways become deregulated in cancer and, accordingly, abnormal PRC expression and/or function have been described in many human malignancies. These studies have also shown that PRCs impact on pathways pertinent to hallmarks of cancer, including enhanced proliferative capacity, suppression of apoptosis and cellular senescence programs, and enhanced invasive potential [4].

Herein, we summarize the existing evidence linking PRC-mediated epigenetic aberrations to malignancy, with a particular focus on established links between PRC2, genomic instability and cancer.

## 2. Polycomb Repressor Complex 2 Is Deregulated in Malignancy

Both loss and gain-of-function mutations in PRC2 components have been identified in human malignancies (Table 1), highlighting their complex roles in tumorigenic processes.

Germline mutations in *EZH2*, *EED* and *SUZ12* have been implicated in Weaver syndrome (MIM# 277,590), a rare, multisystem genetic disorder characterized by prenatal or postnatal overgrowth, limb deformities, and variable degrees of intellectual disability and facial features [70,71,72]. In vitro studies have shown that these mutations cause a decrease in H3-K27 methylation, which suggests a causative link between loss-of-function mutations of PRC2 components, chromatin remodeling, and Weaver syndrome pathology [72,73]. Intriguingly, among Weaver syndrome patients, there is a relatively increased risk of childhood neuroblastoma [74,75]. Moreover, *SUZ12* at 17q11.2 is mapped approximately 560 kb downstream of the neurofibromatosis type 1 (*NF1*) gene, and approximately 5–10% of NF1 patients exhibit microdeletions that encompass both *NF1* and *SUZ12* [76]. This subgroup of NF1 patients present a much higher burden of neurofibromas compared with patients lacking such deletions [76]. Mutations in *SUZ12* and *EED* that lead to reduced H3-K27me3 have also been described in malignant peripheral nerve sheath tumors, which are a type of aggressive sarcomas [77]. Beyond malignancies of the nervous system, loss-of-function point mutations and deletions in *EZH2* are detected in patients with myelodysplasia [51], a bone marrow failure syndrome that is regarded as an “epigenetic disease” [78]. Mice with conditional deletions of *EZH2* and *TET2* in hematopoietic stem cells develop myelodysplastic syndrome and myeloproliferative neoplasms [79].

In contrast, numerous epithelial and hematological malignancies possess elevated expression and gain-of-function mutations in PRC2 components. For example, monoallelic missense mutations of Tyr641 and Ala677 residues of EZH2 occur in more than 22% of diffuse large B-cell lymphoma (DLBCL) and follicular lymphoma [54]. Whereas the disease-associated Tyr641 mutations display limited ability to perform the monomethylation reaction of H3-K27 compared to wild-type EZH2, the mutated protein has enhanced catalytic efficiency for the subsequent mono- to di- and di- to trimethylation reactions [80]. As a result, the simultaneous production of a Tyr641-mutated and a wild-type protein in DLBCL leads to an overall exaggerated EZH2 activity [80,81].

EZH2 is also upregulated with disease progression in chronic lymphocytic leukemia (CLL), and its levels are higher in the most aggressive subgroup of CLL patients [82]. Similarly, high levels of EZH2 expression correlate with an adverse prognosis in solid tumors, such as prostate [11] and breast cancer [13]. SUZ12 is also overexpressed in malignancies such as mantle cell lymphoma, pulmonary carcinomas and melanoma, in part through gene locus amplification [83]. In the mouse, EZH2 and SUZ12 are activated upon exposure to known environmental carcinogens such as 12-dimethylbenz[a]anthracene (DMBA) [84], arsenic [85], and tobacco smoke [86]. Collectively, these observations are consistent with an oncogenic role for PRC2 deregulation.

How could these opposing associations of PRC2 components with malignancy be reconciled? The identification of both gain- and loss-of-function mutations of PRC2 in cancer may indicate that a critical balance of polycomb activity is essential for cellular homeostasis, with either loss or gain of PRC function being potentially tumorigenic. PRCs may also promote or suppress carcinogenesis, depending on the cellular context. Context-dependency may reflect the impact of other cellular factors on PRC complex assembly or function. It is known, for example, that the kinase Akt, which is frequently activated in certain tumor types, mediates phosphorylation of EZH2 at Ser21, leading to its reduced affinity for histone H3, reduced H3-K27me3 and consequent derepression of EZH2-silenced genes [87]. Conversely, the net outcome of PRC activation on chromatin structure and transcriptional responses may depend on the activity of additional signaling molecules. For example, polycomb-like PRC2-associated factor PHD finger protein 1 (PHF1) interacts with EZH2 and modulates its activity in favor of the repressive H3-K27me3 mark [88].

PRC2-independent functions of EZH2 have also been described, adding further complexity in the functional effects of disease-associated EZH2 mutations. Of note, EZH2 physically interacts with and supports the constitutive activation of NF-κB target gene expression in estrogen receptor (ER)-negative breast cancer cells independently of its histone methyltransferase activity [89]. However, EZH2 acts in an opposite manner in ER-positive luminal-like breast cancer cells and represses NF-κB target gene expression by interacting with ER and directing repressive histone methylation on the promoters of NF-κB target genes [89].

Given the complex associations between malignancy and PRC deregulation across tumor types, evidence for causative links has been sought, and efforts have been made to gain insight into the mechanisms by which PRC components influence malignant transformation and growth.

## 3. Mechanisms by Which PRC2 Influences Malignant Transformation and Growth

PRCs may affect malignant transformation by at least three mechanisms: (a) by regulating tumor suppressor gene (TSG) expression; (b) by influencing the DNA damage response (DDR) and, (c) by impacting on the fidelity of DNA replication.

### 3.1. Regulation of Tumor Suppressor Gene Expression by PRC2

The functional deregulation of PcGs may indirectly promote genomic instability through the modulation of TSG expression. The most well-studied TSG targets of PcGs are the CDKN2A and CDKN2B loci encoding p14 (ARF), p15 (INK4B) and p16 (INK4A), whose products participate in major tumour suppressor networks that are disabled in human cancer.

It has been shown that in normal diploid cells, the nascent long non-coding RNA*ANRIL* is transcribed at the transcription start site in the *p16^INK4A^* gene, and associates with SUZ12 to assemble a PRC2 complex that initiates H3-K27me3. In turn, *ANRIL* and PRC2 recruit PRC1, providing another docking site to bind H3-K27me3 and induce H2A-K119Ub, which results in epigenetic repression of the *p16^INK4A^*, *p15^INK4B^*, and *ARF* genes [90,91]. In aged or stressed cells, *ANRIL* and EZH2 levels decrease, the latter through activated p53 [92], causing disruption of the PRC2 complex and loss of H3-K27me3 along the INK4B and INK4A-ARF loci. Without this methylation mark, the PRC1 complex is displaced, leading to progressive changes in chromatin around the INK4B and INK4A-ARF loci and access to specific transcriptional activators [91]. The ensued induction of *p16^INK4A^*, *p15^INK4B^*, and *ARF* gene expression results in senescence.

The exact mechanism by which PRC deregulation impacts on epigenetic control of the INK4B and INK4A-ARF loci in malignancy remains nebulous and may involve various overlapping pathways. Thus, overexpressed EZH2, that mimics the elevated levels of EZH2 frequently found in cancer, leads to permanent transcriptional repression of those loci, thereby inhibiting senescence and allowing proliferation under conditions of oncogene or genotoxic agent-induced stress [93]. Other studies point to a major role of the chromodomain-containing protein CBX8, which is a component of a PRC1 complex, in this effect. CBX8 is over-expressed in various types of cancer [94], and experimental data show that its ectopic expression leads to repression of the INK4A-ARF locus, bypass of senescence and induction of cellular immortalization [95]. Deregulated expression of EZH2 and CBX8 may cooperate to modulate *p16^INK4A^*, *p15^INK4B^*, and *ARF* gene expression, as indicated by their synergy in driving lymphomagenesis in the mouse [96]. Further, elevated levels of *ANRIL* are detected in various types of cancer, including those of stomach, lung and liver, and may also contribute to evasion from senescence and inhibition of apoptosis [97].

Another TSG target of EZH2 in multiple cancers is the E-cadherin gene (CHD1), the downregulation of which is critical for epithelial-mesenchymal transition (EMT) and metastasis [46,98,99]. PRC2 binds the promoter of E-cadherin, and EZH2 mediates the repression of E-cadherin transcription by activation of H3-K27me3. Recent reports also suggest an important role for EZH2 in the epigenetic control of prostate cancer through modulation of TSG expression. EZH2 is upregulated in prostate cancer through amplification of the *EZH2* gene [100], deletion of its negative regulator miR-101 [101], and transcriptional regulation by *MYC* [102] and *ETS* gene family members [103]. This, in turn, leads to H3-K27me3-associated silencing of tumor suppressor genes and developmental regulators such as DAB2IP, MSMB, SLIT, TIMP-2, and TIMP-3, which promote a dedifferentiation program, thereby maintaining a stem cell-like state [104,105] and contribute to the increased growth, proliferation and invasive phenotype of prostate cancer cells.

### 3.2. Regulation of DNA Damage Response (DDR) by PRC2

PcG genes directly and indirectly regulate aspects of the DDR, which functions as a barrier to genomic instability and cancer [106,107,108,109]. The DDR utilizes proteins involved in sensing, signaling and the repair of DNA lesions caused by genotoxic agents or oncogene-induced replication stress. Recent work has unveiled the involvement of specific PcG proteins in DDR. For instance, overexpression of EZH2 in human mammary epithelial cells causes a drastic decrease in the expression of multiple RAD51 paralogs (RAD51B/RAD51L1, RAD51C/RAD51L2, RAD51D/RAD51L3, XRCC2, and XRCC3) required for homologous recombination (HR) double-strand break (DSB) repair. As a result, EZH2 overexpression leads to a significant decrease in the numbers of DNA repair foci, increased aneuploidy, and a reduced survival rate of cells exposed to genotoxic stress [14]. Other core PRC2 components, such as SUZ12, PHF1 and the H3-K27 methyl mark, have also been detected at sites of DNA damage, suggesting that the entire PRC2 complex is present at DSBs [110,111,112,113].

The observation that the PRC2-associated PHF1 rapidly associates with DSBs in a KU70/KU80-dependent manner is pertinent to the mechanism by which PRC2 is recruited to sites of DNA damage. Knockdown of PHF1 increases the frequency of homologous recombination, and sensitizes cells to irradiation. The direct physical interaction of PHF1 with KU70/KU80 proteins strongly suggests that this PRC2 protein promotes the non-homologous end joining (NHEJ) repair pathway. Besides Ku70/Ku80, PHF1 is also associated with Rad50, DHX9, SMC1 and p53 proteins, which are all involved in the response to DSBs and other genome maintenance mechanisms, including HR repair [111]. The possibility that PRC2 participates in DSB repair is further supported by studies showing that EZH2 and SUZ12 interact with the poly (ADP-ribose) polymerase 3 (PARP3), a protein known to associate with components of the NHEJ repair pathway [114]. Campbell et al. [115] also found that PRC2 proteins are recruited with kinetics similar to other early DSB repair proteins, and that PARP activity is required for retaining PRC2 at sites of DNA damage. Furthermore, depletion of EZH2 decreases the efficiency of DSB repair, thereby increasing the sensitivity of cells to γ-irradiation [115].

In addition to acting directly at sites of DNA damage, PRC2 participates in several other aspects of the DDR. Thus, EZH2 has been implicated in the activation of cell cycle checkpoint regulators, such as the cyclin-dependent kinase inhibitor p21 and Chk1/2 in p53-proficient or p53-deficient cancer cells, respectively [116]. The importance of this finding is highlighted by the fact that EZH2 depletion results in abrogation of both G1 and G2/M cell cycle checkpoints, thereby directing the DDR toward apoptosis and increased sensitivity to genotoxic agents, such as the chemotherapeutic drugs etoposide and doxorubicin [116].

EZH2 also affects the intracellular localization of BRCA1, a protein that regulates DNA repair, activation of cell-cycle checkpoints, and chromosomal stability. Conditional EZH2 overexpression in normal mammary epithelial cells was found to induce Akt1-dependent nuclear export and cytoplasmic retention of BRCA1, and increase the levels and catalytic activity of Aurora kinases A and B, which mediate centrosome maturation, separation and spindle formation. As a result, the upregulation of EZH2 led to aberrant mitoses, polyploidy and genomic instability [117].

### 3.3. PRC2 Impacts on the Fidelity of DNA Replication

Recent findings directly implicate PcG activity in DNA replication and genome maintenance. Piunti et al. [118] reported that PcG proteins associate with replication forks, and that the loss of EZH2 delays the progression of DNA replication and leads to the accumulation of a greater number of asymmetric and unidirectional DNA replication forks. DNA replication stalling can trigger firing of dormant origins and may activate the DDR; as a result, the ablation of EZH2 in fibroblasts leads to an increased number of 53BP1 foci, which are a marker of damaged DNA, compared with wild-type cells. These effects are associated with reduced cell proliferation, even in the absence of functional INK4A/ARF-pRb-p53 tumor suppressor pathway. Moreover, the absence of *ezh2* suppressed H-RAS^V12^ or MYC-induced fibroblast transformation and oncogenic potential. Thus, in the absence of functional checkpoints, cells do not undergo a cell cycle arrest, but their proliferation is still be dependent on EZH2 activity [118].

## 4. Pharmacological and Dietary Interventions Targeting EZH2 Deregulation in Cancer

Moderate caloric restriction without malnutrition inhibits spontaneous, chemically-induced or transplanted tumors in several models of cancer in the mouse and non-human primates [60]. The mechanisms responsible for these beneficial effects include decreased production of growth factors and anabolic hormones, decreased plasma concentrations of inflammatory cytokines, enhanced anticancer immunosurveillance, reduced synthesis of reactive oxygen species and thus, DNA damage induced by free radicals [119,120,121]. Conversely, epidemiological studies show that obesity elevates the risk of cancer of the colon, liver, pancreas, and esophagus, and experimental data link excess adiposity to genomic instability, tumor initiation and progression [122,123].

Accumulating evidence suggests that diet may impact on histone modification processes through PcG proteins, which adds another dimension to the prevention and management of obesity-propelled cancer. This is perhaps best exemplified by the observation that obesogenic stimuli can trigger EZH2-mediated H3-K27me3 of Wnt genes, which physiologically function as inhibitors of adipogenesis [124]. The ensued suppression of Wnt expression leads to upregulation of the transcription factors PPARγ and C-EBPα, which promote adipogenesis and obesity [125]. Conversely, low protein intake is highly effective in inhibiting tumor growth in human xenograft prostate and breast cancer models by reducing expression of EZH2 and histone mark H3-K27me3 [126]. Moreover, *O*-GlcNAc glycosylation has been found to enable PcG repression [127,128]. The abundance of *O*-GlcNAc is dependent on the availability of glucose. The requirement of glycosylation for effective PRC-mediated repression would therefore make this mechanism highly dependent on an energy-rich diet [129], thus connecting nutrition-influenced epigenetic reprogramming to the etiology of metabolic diseases and cancer [130].

Dietary components such as ω-3 PUFAs and antioxidants are known to confer antitumor properties, which have recently been linked to EZH2 regulation. Thus, ω-3 PUFAs post-translationally control the turnover of EZH2 through ubiquitination and proteasomal degradation of an EZH2 pool [131]. Antioxidants may play an important role in modulating PcG action. Mice lacking the PRC1 subunit Bmi1 develop numerous abnormalities, including a severe defect in stem cell self-renewal, and a shortened lifespan. The phenotypic outcomes of Bmi1 ablation are largely alleviated after treatment with the antioxidant *N*-acetylcysteine, pointing to an unexpected role of PcGs in maintaining mitochondrial function and redox homeostasis [132]. In line with these findings, the expression of EZH2 and the levels of EZH2-mediated H3-K27me3 are reduced in cancer cell cultures exposed to natural antioxidants such as resveratrol, a polyphenolic flavonoid present in red grape [133]; curcumin, a member of the ginger family [134,135,136,137]; epigallocatechin-3-gallate, a green tea catechin [138,139]; and other phytochemicals (reviewed in Shahabipour et al. [140]).

The aforementioned observations, coupled with the elevated expression levels and catalytic activity of PRC2 components in aggressive solid tumors and hematopoietic malignancies, raised the possibility that EZH2, EED and/or SUZ12 could serve as pharmacological targets in cancer (Table 1). Along these lines, McCabe et al. developed GSK126 as a potent small-molecule inhibitor of EZH2 methyltransferase activity, which decreases global H3-K27me3 levels and reactivates silenced PRC2 target genes in lymphomas bearing gain-of-function EZH2 mutations [141]. GSK126 was found to effectively inhibit the proliferation of DLBCL cell lines and markedly inhibit the growth of mutated EZH2-bearing DLBCL xenografts in mice.

DZNep (3-deazaneplanocin A) is an *S*-adenosyl-homocysteine hydrolase inhibitor that blocks the EZH2-associated H3-K27me3 and reactivates PRC2-silenced genes to induce apoptosis and amplify the DDR and cytotoxic effects of chemotherapy in malignant, but not normal, cells [116,142]. Together, these data demonstrate that the pharmacological inhibition of EZH2 activity may provide a promising treatment for lymphomas and solid tumors bearing exaggerated EZH2 methyltransferase activity. The targeting of other proteins in the complex, such as EED and SUZ12, also holds promise as potential anticancer strategy by disrupting proper PRC2 complex assembly [143].

## 5. Conclusions

PRC2 displays complex roles in tumorigenic processes, as highlighted by the identification of both loss and gain-of-function mutations affecting PRC2 components in human malignancies (Table 1). These observations may reflect context-dependent cancer-promoting inputs, or the requirement for a critical balance of polycomb activity for cellular homeostasis. This is exemplified by the observation that whereas mutational loss of EZH2 promotes the development of myelodysplastic syndrome (MDS), it attenuates MDS progression to acute myeloid leukemia by suppressing expression of the leukemic oncogene *Hoxa9* [144]. To explain the opposing roles of PRC2 in cancer, Comet et al. [145] proposed that PRC2 sets a threshold for gene activation, with deregulated PRC2 promoting epigenetic instability. This instability leads to transcriptional deregulation, thereby increasing the risk of cancer development. The composition of PRCs in defining suppression vs activation states of target genes needs also to be better understood in different cellular contexts.

The gain-of-function contributions of PRC2 to cancer are expanding, and include the repression of the INK4A/ARF locus and other TSGs, a reduced capacity for DNA damage repair, direct effects on DNA replication, and the transcriptional deregulation of developmental regulators that promote a de-differentiation program. These molecular changes are associated with defects in checkpoint activation, aberrant mitoses, polyploidy and genomic instability. However, further work is needed to fully appreciate the mechanisms by which PRC2 rewires DNA damage response and tumor suppressor gene networks to enable normal cells to escape antitumor barriers leading to genomic instability and cancer. EZH2 may also have PRC2-independent roles, including serving as a transcriptional coactivator of transcription factors such as NF-κB. The contribution of this non-canonical function to the role of EZH2 in oncogenic transformation also requires additional studies.

Given the evidence for the enzymatic gain-of-function of EZH2 in various tumor types, the development of pharmacological and dietary interventions affecting EZH2 has been an active area of investigation for the prevention and management of human malignancies. Currently, compounds that either target the catalytic activity of EZH2, or disrupt the PRC2 complex, are under development and validation in clinical trials. However, the contextual role of PRC2 in cancer, coupled with evidence indicating the development of resistance to such compounds [146], emphasizes the need for the identification of specific biomarkers that can predict beneficial outcomes of treatment with PRC2 inhibitors. Of relevance, a recent study has shown that the overexpression of EZH2 may in fact be a consequence, rather than a cause of certain malignancies, and that whereas the high expression of EZH2 is correlated to poor prognosis in breast cancer, this association stands only when EZH2 expression couples to proliferation [147]. In contrast, the proliferation-independent expression of EZH2 displays an inverse association with tumor outcome, with low EZH2 expression being linked to poor prognosis [147]. These findings underscore the need for an in-depth understanding of the molecular intricacies underpinning the tumor-associated effects of EZH2, and the thorough assessment of its putative utilization as a target for cancer therapy.

## Figures and Tables

**Table 1:** Polycomb repressor complex 2 status in cancer.

PRC2 Status	Associated Cancer Type	Experimental Therapeutic Approaches
EZH2 overexpression	Hematological malignancies [5]	miR-22 [6]
	Pancreatic cancer [7,8]	DZNeP with gemcitabine [9]
	Chronic Pancreatitis [10]	
	Prostate cancer [11]	MicroRNA-101 [12]
	Breast cancer [13,14]	
	Bladder carcinoma [15,16,17]	NSC745885 [18], Gambogic acid and methyl jasmonate [19]
	Gastric cancer [20]	GSK126 [21]
	Lung cancer [22,23]	JQEZ5 [24], Schlafen11 [25]
	Hepatocellular carcinoma [26,27,28]	GSK343 [29,30]
	Glioblastoma multiforme [31]	DZNep, shRNA [32]
	Cervical Cancer [33,34]	
	Ovarian cancer [35]	
	Melanoma [36]	GSK126 [37], GSK503 [38]
	Soft Tissue Sarcoma [39,40]	
	Lymphoma, Mantle-Cell [41]	
	Colorectal cancer [42,43,44]	
	Retinoblastoma [45]	
	Tongue cancer [46,47]	
EZH2 loss of function mutation	Peripheral nerve sheath tumors (MPNST) [48]	
	Myeloproliferative neoplasms (MPNs) [49]	
	Myeloid –myelodysplastic malignancies [50,51]	
	Pediatric tumors of the central nervous system [52]	
	T cell acute lymphoblastic leukemia [53]	
EZH2 gain of function mutation	Lymphomas EZH2-Tyr641 [54] EZH2-Ala677 [55] & Ala687 [56]	Small molecular inhibitor EI1 [57], GSK126 [58], EPZ005687 [58]
EED overexpession	Breast cancer [59]	
	Colorectal cancer [60]	
	Hepatocellular carcinoma [61]	Wedelolactone [62]
EED loss of function	Adenosquamous lung tumors [63]	
	Malignant peripheral nerve sheath tumors [64]	
SUZ12 overexpession	Breast cancer [61]	
	Colorectal cancer [60,61]	
	Hepatocellular carcinoma [61]	
	Ovarian cancer [65]	
	Gastric Cancer [66,67]	
	Non-small cell lung cancer [68]	
	Bladder cancer [69]	
SUZ12 loss of function	Malignant peripheral nerve sheath tumors [64]	
	T cell acute lymphoblastic leukemia [53]	
